# Secondhand smoke and e-cigarette aerosol exposure in dogs and cats: A scoping review with implications for smoking cessation among pet owners

**DOI:** 10.18332/tpc/217790

**Published:** 2026-04-16

**Authors:** Matteo Cerquetella, Vincenzo Zagà, Valentino Gardi, Andrea Marchegiani, Claudia Vitturini, Maria Sofia Cattaruzza

**Affiliations:** 1 School of Biosciences and Veterinary Medicine, University of Camerino, Camerino, Italy; 2 Independent Researcher, Bologna, Italy; 3 Department of Legal Studies, Department of Civil, Chemical, Environmental, and Materials Engineering, Alma Mater Studiorum-University of Bologna, Italy; 4 Department of Public Health and Infectious Diseases, Sapienza University, Rome, Italy

**Keywords:** pets, secondhand smoke, electronic cigarette, motivation to quit, one health

## Abstract

**INTRODUCTION:**

The harmful effects of secondhand smoke (SHS) on human health are well established, whereas its impact on pets remains underexplored and less widely recognized by both the scientific community and the general public. This scoping review aimed to synthesize current evidence on the health effects of SHS and electronic cigarette (e-cigarette) aerosol exposure in dogs and cats.

**METHODS:**

An in-depth PubMed search was conducted using predefined keywords, limited to free full texts in English with available abstracts and relevance to the topic.

**RESULTS:**

Twenty-six eligible articles were analyzed. Of the 11 studies investigating cancers, respiratory, and cardiovascular diseases in dogs and cats, over half reported statistically significant associations with SHS. Biomarkers such as cotinine were identified in serum, urine, fur, and amniotic fluid, confirming that pets are not only passive recipients of human environmental exposures but may also serve as environmental sentinels within a One Health framework. No eligible studies addressed e-cigarette aerosol in pets, but human data indicate potential risks. Notably, awareness of SHS risks to pets may motivate owners to adopt smoke-free behaviors.

**CONCLUSIONS:**

Despite some inconsistencies, available evidence supports the harmful effects of SHS in pets. Veterinary and public health professionals can leverage these findings to raise awareness and promote smoking cessation, integrating pet welfare into tobacco control strategies. Further studies are needed to standardize exposure assessment and evaluate long-term health effects.

## INTRODUCTION

Dogs and cats can be exposed to cigarette smoke (CS) in various ways, in the past mainly in experimental contexts and nowadays through passive smoking, the so-called secondhand smoke (SHS). SHS is inhaled involuntarily by subjects who come into contact with ‘active’ smokers, and it is the main pollutant in closed environments^1^. It is the result of the smoke exhaled by the active smoker, added to the smoke produced by the slow and imperfect combustion (300–500°C) of the cigarette left to burn in the ashtray or in the hand between one puff and the next. SHS contains, albeit in different percentages, the thousands of toxic substances found in active smoking^1,2^. Pets are also exposed to thirdhand smoke, which consists of particulate material that accumulates on carpets, curtains, clothes, and other surfaces^3-5^.

The toxicity of tobacco smoke is linked to the products of combustion. During the inhalation phase, tobacco burns at about 800–1000°C, releasing thousands of substances toxic to health in a mixture of gases and particles. Then the smoke partially and gradually cools before reaching the oropharynx and the bronchopulmonary system of the smoker. Its composition is not constant, and its nature varies depending on the type of tobacco, the drying method, the fertilizers and pesticides applied, the additives used in the manufacture of the product, etc. Among molecules constantly found in the smoke, there are nicotine, tars, and flavoring agents, but also toxic gases such as carbon monoxide, nitrogen oxide, hydrocyanic acid, ammonia, and heavy metals like cadmium, lead-210, polonium-210, chromium, and mercury. Furthermore, the advent of new and more sensitive chromatographic analytical techniques has allowed the Food and Drug Administration (FDA) to update the number of different substances released by the combustion of tobacco, bringing them to over 7 thousand^2^, some of which have irritant properties (e.g. nicotine and free radicals) and others are recognized as carcinogenic (e.g. 4-aminobiphenyl, benzopyrene, toluene, dimethyl-nitrosamine, polonium 210)^2,6,7^. While in 1964, the ‘Surgeon General Report on Smoking and Health’ or ‘Terry Report’^8^ declared that tobacco smoking was causally associated with lung cancer and then evidence emerged showing its association with many other diseases, it was only in 2004 that SHS has been classified as a carcinogen by the World Health Organization’s Agency for Research on Cancer^9^, finally defeating tobacco industry attempts to obstruct the publication of this monograph to prevent increased smoking restrictions^10^. Again, scientific evidence in human medicine has demonstrated how SHS was causally associated with many other diseases, and it is now also demonstrating how active and passive exposure to emissions of electronic cigarettes can produce different types of harm^11-13^. Indeed, albeit e-cigarettes are claimed as safer than traditional smoking, their aerosol contain metals, toxic and carcinogenic molecules and a growing body of evidence in man has been showing how their use negatively impact both cardiovascular and respiratory systems, causing elevated blood pressure, arterial stiffness, vascular endothelial dysfunction, enhanced angiogenesis, cardiorenal fibrosis, accelerated atherosclerotic plaque development, elevate levels of type 2 inflammatory cytokines such as IL-4, IL-5, and IL-13, increased cellular oxidative stress, increased airway reactivity, airflow obstruction, airway inflammation, and emphysema. Also, DNA methylation changes linked to carcinogenesis have been demonstrated^11-13^. Additionally, in human medicine, secondhand nicotine vape exposure was always associated with increased risk of bronchitis symptoms and shortness of breath among young adults^13^.

Despite a growing awareness and the introduction of some regulations, SHS is still perceived as less harmful, although there is no safe level of exposure. Worldwide, it causes more than 1.2 million premature deaths each year, serious cardiovascular and respiratory diseases are attributable to it, and it is estimated that almost half of children regularly breathe air polluted by tobacco smoke in public places, and 65000 die each year from illnesses attributable to SHS^14^. *De facto*, the passive smoker risks most of the diseases to which the active smoker is prone. Exposure to SHS is characterized by a dose-response effect: the more intense and prolonged the exposure to environmental smoke, the greater the consequences resulting from the harmfulness of the exposure, and this is related to both acute and chronic conditions as well as to the possible development of neoplasms^14^. Exposure to SHS alters the immune mechanisms during allergic diseases; it compromises the responses of the bronchial epithelium by modifying the expression and activation of innate immunity receptors and determines the destruction of desmosomes and tight junctions with increased permeability to allergens, pollutants, and infectious agents and the release of inflammatory mediators (IL-8, IL-1β, sICAM-1)^15^.

In pregnancy, SHS causes complications and low birth weight; for newborns, it increases the likelihood of sudden infant death syndrome, while in childhood, it leads to higher rates of respiratory and middle ear infections, meningococcal infections, and asthma^8,16-18^.

The harms of passive smoking are particularly significant for true ‘involuntary’ passive smokers, namely, children and pets. Pets play an important role in society, which is especially relevant nowadays as the world’s population ages. Their role is associated with benefits to physical and mental health^19,20^. In Italy, in 2025, 40.5% of Italians declared that they are hosting an animal, and in 2024, the pet population was 65 million animals (in particular, dogs and cats together reached a number of approximately 21 million)^21,22^. Similar figures are reported for the United Kingdom, where pet ownership peaked to a level of 59% in 2020–2021 (with an estimated 12 million dogs and 12 million cats living in homes)^23^.

This scoping review aims to update knowledge on the effects of SHS and e-cigarette aerosol on dogs and cats, which in many cases are actual family members, in order to map the current state of research and identifying possible knowledge gaps and to raise pet owners’ awareness of the health potential risks they expose their pets to, as a possible motivational incentive for owners to quit smoking.

## METHODS

Within this scoping review and to identify relevant articles, the PubMed Database was used. No time limits were placed on the search, which ended on 16 February 2025, and all types of studies were considered. One of the researchers (MC) searched the following key words: ‘passive smoke dog’; ‘passive smoke cat’; ‘passive smoke pets’; ‘smoke dog’; ‘smoke cat’; ‘smoke pets’; ‘passive smoking dog’; ‘passive smoking cat’; ‘passive smoking pets’; ‘second-hand smoke dog’; ‘second-hand smoke cat’; second-hand smoke pets”; ‘environmental tobacco smoke dog’; ‘environmental tobacco smoke cat’; ‘environmental tobacco smoke pets’; ‘e-cigarette dog’; ‘e-cigarette cat’; ‘e-cigarette pets’.

The search was duplicated and conducted blindly by another researcher (MSC) who used the following string: (((smok*) OR (second-hand smoke) OR (passive smok*) OR (Environmental tobacco smoke)) AND ((cat) OR (cats) OR (dog) OR (dogs) OR (pet) OR (pets))).

Both researchers independently eliminated duplicates, articles not available in ‘free full text’ (PubMed text availability search filter), articles without abstract (PubMed text availability search filter), and articles in languages other than English (PubMed article language search filter). After having carried out an initial selection of the contents based on the abstracts, studied the full text of all the articles deemed suitable, and compared for doubtful cases, the two authors agreed to include 26 articles grouped according to their main topics in the next paragraphs.

**Figure 1:**
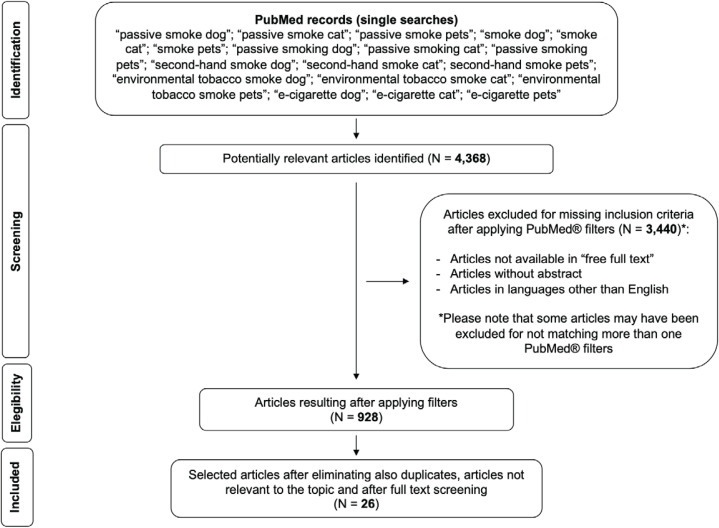
PRISMA flow diagram of search strategy

In order to widen the knowledge on available literature and provide more comprehensive overview and support the broader objectives of the present review, some articles that did not meet the formal inclusion criteria - due to lack of open-access free full text (as per the PubMed text availability search filter) and/or not in English, and/or present in different databases - were nonetheless analyzed in a specific paragraph (see section ‘Further relevant evidence on passive smoke and pets’). Figure 1 reports the PRISMA flow diagram of the search strategy.

## RESULTS

### Dogs and cats in experimental studies

With the above-mentioned search criteria and keywords, the earliest studies retrieved involved dogs and cats as experimental models; these articles date back to the 1970s, and they do not go beyond the 1980s (Table 1).

**Table 1:** Studies involving dogs and cats in an experimental context (International PubMed free research service for biomedical scientific literature)*

*Study number*	*Animals (n)*	*Reference*
**1**	cats (-)	[24]
**2**	cats (33)	[25]
**3**	dogs (69)	[26]
**4**	dogs (-)	[27]
**5**	dogs (12)	[28]
**6**	dogs (10)	[29]

* Last access 16 February 2025. Note: ‘-’ stands for ‘absence of information about the number of animals’.

The first study included is from Hall et al.^24^ who found that cigarette smoke may have effects on the central nervous system of cats, in particular causing cortical activation and behavioral arousal. Then the study from Rink et al.^25^ started from the initial assumption that nicotine administration in experimental animals increases cardiac work, blood flow, and oxygen consumption, but at the same time, tobacco smoke contains carbon monoxide, thus possibly reducing tissue oxygenation. To better investigate these effects, 33 anesthetized cats were given nicotine, tobacco smoke, and carbon monoxide and studied their effects on myocardial oxygen tension, confirming the ability of the first two to increase blood pressure and myocardial oxygen tension and that of the third molecule to reduce it^25^. The effect of cigarette smoke was also studied in the anesthetized dog, finding that a short-term exposure can reduce the mucociliary clearance action, to an extent related to the amount of smoking consumed^26^. It was also shown in dogs that CS can decrease the active ion transport and therefore the electrical potential difference across the tracheal epithelium, altering the respiratory tract fluidity and consequently affecting the mucociliary clearance^27^. Another study on the effects on the respiratory apparatus in dogs showed that direct cigarette smoke inhalation caused interstitial fibrosis of the interalveolar septa and destruction of the alveolar walls, leading to emphysema^28^. In dogs, it was found that the inhalation of cigarette smoke condensate constituents can induce pneumonitis, the formation of thrombi in branches of pulmonary vessels, and acalculous cholecystitis^29^.

### Effects of tobacco smoke on dogs and cats as patients

The first studies introducing the evaluation of the effects of smoking on dogs and cats in their connotation of patients, as a consequence of living with smokers, date back 20 years. Among those retrieved, three studies investigated the clinical signs of respiratory diseases in dogs and cats, six studied the possible association between SHS and neoplastic diseases (oral squamous cell carcinoma, lymphoma, lung cancer, and urinary bladder cancer), one was on myxomatous mitral valve disease, and the last one on oxidative stress (Table 2). Of these 11 studies, 3 reported no statistically significant association with SHS, 1 found an unclear relationship; 6 reported a positive association, and 1 suggested that SHS exposure may lead to epigenetic modifications. Among the studies which faild to identify a positive association, the one by Hawkins et al.^30^, designed to investigate risk factors for chronic cough in dogs, found that exposure to passive smoking was higher in dogs with chronic cough than in controls, but the difference was not statistically significant; the 115 dogs with chronic cough included in the study were diagnosed with different diseases and in 6 cases a pulmonary neoplasia was diagnosed^30^. Also, a retrospective case-control study searching for possible associations between SHS and primary lung cancer in dogs failed to highlight this association^31^. All diagnoses of lung cancer were laboratory confirmed, but dogs with histiocytic sarcomas or squamous cell carcinomas were excluded from the analysis due to small numbers. Two control groups were enrolled, one consisting of dogs with mast cell tumors and the other with neurologic diseases^31^.

**Table 2:** Characteristics of studies involving dogs and cats as patients; articles are presented by type of animal investigated (dog, cat, dog & cat); then by type of disease (respiratory, cancer, other) (International PubMed free research service for biomedical scientific literature)*

*Study number [ref] Year Country*	*Condition/disease investigated*	*Type of study*		*Sample size*		*Exposure*		*Results*			*Associations with SHS Remarks*
**DOG**											
1 [30] 2010 USA	Chronic cough (CC) lasting ≥2 months	Cross-sectional comparative study (cases enrolled between 2000 and 2009; controls enrolled between 2005 and 2009)		219 dogs, 115 cases with a cough lasting ≥2 months, and 104 controls with problems other than cough		Dog owners completed questionnaires. Lack of questionnaires for control dogs from the years 2000–2004		SHS exposure (2005–2009): 32% cases (19/59) vs 24% controls (26/109; p=0.243). For 2000–2009, SHS exposure among cases was 41% (47/114)			No statistically significant association Analysis of SHS exposure limited by missing control group questionnaires (2000-2004)
2 [38] 2015 Japan	Chronic bronchitis (CB)	Cross-sectional comparative study assessing concentrations and methylation rates of free-floating DNA fragments in bronchoalveolar lavage fluid (BALF)		19 dogs, all with CB, 8 exposed and 11 not exposed to SHS, recruited between 2005 and 2010. Clinical and laboratory confirmed diagnosis		Dogs with CB classified as SHS-exposed or non-exposed based on serum cotinine values, measured with Cotinine Passive Smoking ELISA Kit		SHS-exposed dogs had higher median total nucleated cells (600 vs 124/μL; p<0.05) and macrophages (438 vs 56/μL; p<0.05). No overall difference in DNA concentration/methylation, but 3/8 exposed dogs showed high BALF DNA methylation			SHS exposure leads to epigenetic modifications of cellular components in BALF
3 [31] 2018 USA	Primary lung cancer (PLC). Dogs with histiocytic sarcomas or squamous cell carcinomas were excluded from the analysis due to small numbers	Retrospective case-control study (review between 2002 and 2012 clinical records and mail survey to dog owners)		470 dogs. PLC cases=135; control-group 1 (mast cell tumor, no other neoplasia)=169; control-group 2 (neurologic disease, no neoplasia)=166. All diagnoses were histopathologic, cytologic, or postmortem confirmed		Passive smoke exposure assessed by number of household smokers (definition of smoker=person who smoke ≥1 cigarette/day, most days/week, in past 5 years prior to the pet’s diagnosis), smoking frequency, indoor smoking allowed (yes/no), tobacco type (cigarettes, cigars, pipe), number of cigarettes smoked per day (0, 1–19, >20) overall and indoors, and dog’s proximity to smokers (lap, next to, same room, absent)		Multivariable logistic regression: any smokers in household OR=0.8 (95% CI 0.4–1.4; p=0.38); any indoor tobacco smoke OR=0.7 (95% CI 0.3–1.6; p=0.41)			No statistically significant association Retrospective study with potential recall/response bias and possible exposure/case misclassification. Passive smoke exposure is higher in controls vs cases, but not statistically significant. Questionnaires mailed to 1178 dog owners (5-year exposure history); 470 (39.9%) returned and included in analysis
4 [3] 2022 USA	Urinary bladder cancer Specifically, urothelial carcinoma (UC)	Nested case-control study		120 dogs were recruited for a bladder cancer screening study, with comparisons made between dogs that did (n=32) or did not (n=88) develop UC during the 3 years of screening		Dog owners answered a questionnaire investigating environmental and host factors. Pack-years for each smoker in the household were combined for each year of the dog’s life Cotinine concentrations (≥0.04 ng/mL) were detected in 51 of 91 dogs with urine samples		Median exposure among smoker-household dogs: 10 pack-years (0.75–36.0) in UC cases vs 1.5 (0.25–7.0) in controls (p=0.052). Multivariable model: living with smokers OR=6.34 (95% CI 1.16–34.69; p=0.033) UC developed in 18/51 dogs (35.3%) with quantifiable cotinine vs 6/40 (15.0%) without (p=0.0165)		Statistically significant association Cohort approach and use of biomarkers to assess exposure	
5 [35] 2017 Portugal	Lymphoma	Immuno-cytochemical study including subtyping and cell proliferation analysis		23 dogs, 11 smoker owners, 8 non-smoker owners, and 4 owners not reporting smoking status		Dog owners completed a questionnaire on tobacco smoke exposure: household smokers (former/current), daily cigarette consumption, and cumulative smoking duration over the past decade		Ki67 index (lymphomas’ proliferation) positively correlated with smoking status (r=0.753, P=0.002) and number of household smokers (r=0.641, p=0.018). ANOVA: mean % Ki67+ cells higher in smoker-owner group (0.64±0.22) vs non-smoker (0.25±0.19; p=0.011)		Statistically significant association High Ki67 indices correlated with the number of smoker cohabitants	
6 [36] 2025 Portugal	Non-Hodgkin lymphoma (NHL)	Case-control study		113 dogs, 55 cases, and 58 controls Diagnosis of cases confirmed through histopathological or cytological examinations between 2005 and 2016. Owners’ cases contacted via phone, email, or in-person; owners’ controls recruited via online questionnaire sent to University of Porto employees (faculty, staff, admin)		Secondhand and thirdhand smoke exposure assessed via composite ‘smoking index’ (sum of: smokers present/number, mean daily cigarettes per smoker, whole-house smoke presence, dog’s full house access, curtains/carpets, incense use, fireplace, house type)		Dogs with lymphoma had significantly higher mean of smoking index (13.7±12.5 vs 8.4±9.3; p<0.001) and adjusted OR=1.06; p=0.015 No individual tobacco exposure factor reached statistical significance		Statistically significant association	
7 [37] 2014 Denmark Sweden	Myxomatous mitral valve disease (MMVD) confirmed by echocardiography and Endothelial Dysfunction (ED)		Case-control study measuring plasma concentrations of dihydrobiopterin (BH2) and tetrahydrobiopterin (BH4) [a key cofactor for endothelial-derived vasodilator nitric oxide production, it is oxidized to inactive BH2 under oxidative stress] quantified by high-performance liquid chromatography with fluorescence detection		Total 84 dogs: 47 cases and 33 controls (no details on recruitment reported)		Passive smoking was defined as dogs living in a home where the owner smoked indoors		Dogs from homes with smoking had significantly lower plasma BH4 concentrations (no concentration values reported)	Statistically significant association SHS is associated with lower plasma BH4 concentrations, thus reducing endothelium-derived vasodilator nitric oxide	
**CAT**											
8 [33] 2003 USA	Oral squamous cell carcinoma (OSCC)		Prospective case-control study: Cats with a histologically confirmed diagnosis presenting to a large veterinary referral hospital between 1994 and 2000		36 cats with OSCC and 112 renal disease control cats		Cat owners completed a mailed questionnaire on 2-year pre-diagnosis exposures: household smoking (ever/years), number of smokers, total daily cigarette count, and tobacco type (cigarettes, cigars, pipes)		Multivariable logistic regression: cats with owners smoking 1–19 cig/day had statistically significant increased risk (OR=4.00; 95% CI 1.1–14.8; p=0.037) vs cats with non-smoking owners, but no increased risk with ≥20 cig/day (OR=1.3; 95% CI: 0.3–5.5; p=0.77). Other non-statistically significant results: ever lived with a smoker (OR=2.3; 95% CI: 0.8–6.5), living ≥5 years with a smoker (OR=2.7; 95% CI: 0.8–9.0), or living with ≥2 smokers (OR=2.8; 95% CI: 0.6–12.6)	Association not clear Small sample size in some categories	
9 [34] 2022 Italy	Oral squamous cell carcinoma (OSCC)	Prospective observational case-control study. Cats with a cytological or histological diagnosis of OSCC over a 3-year period (2018–2020) were prospectively included		100 cats and 500 healthy controls. Control owners stated cats never had oral tumors. Cats with OSCC were compared with age-matched controls		Cat owners completed anonymous online questionnaire including indoor smoking (yes/no)		Multivariable logistic regression: SHS significantly increases OSCC risk (OR=1.77; 95% CI: 1.05–3.00; p=0.03)		Statistical significant association	
10 [4] 2023 Turkey	Oxidative stress and pro-inflammatory cytokines	Cross-sectional comparative study		40 healthy cats, 20 exposed and 20 not exposed to SHS, recruited between 2018 and 2020		SHS exposure assessed by serum cotinine, measured with cat-specific commercial ELISA kit		In SHS, biomarkers of redox status (total oxidant status, oxidative stress index, protein carbonyl groups, advanced oxidation protein products, lipid hydroperoxides levels), copper level, pro-inflammatory cytokine as interferon gamma and interleukins 1β, 2 and-6 were all significantly higher, whereas, total antioxidant status value and copper-zinc -superoxide dismutase activity were significantly lower		Statistical significant association SHS impaired the oxidant/antioxidant balance, potentially triggering the release of pro-inflammatory cytokines	
**DOG & CAT**											
11 [32] 2018 Taiwan	Various respiratory diseases Definition of ‘respiratory disease’ was: ‘having a clinical diagnosis of a disease involving the respiratory system’	Prospective observational case-control study. Dogs and cats were prospectively recruited between 2016 and 2017		202 animals: 121 dogs, 81 cats. Cases (83 dogs, 64 cats): chronic/current respiratory disease (alone or along with various other diseases). Controls (38 dogs, 17 cats): no current/past respiratory disease (could be either healthy or ill)		Owners completed SHS questionnaire (yes/no; frequency: always/often/occasionally). Household PM2.5 measured in areas where animals spent most time as proxy for indoor air pollution (SHS, incense, cooking)		SHS not significantly associated with respiratory disease in dogs or cats. In cats, household PM2.5 higher in cases vs controls (median 38.6 vs 27.4 μg/m³; p=0.017). In cats, PM2.5 >35 μg/m³ significantly increases respiratory disease risk (adjusted OR=4.13; 95% CI: 1.12–15.27; p=0.03)		No statistical significant association In cats, statistically significant association with PM2.5 Low number of controls and clinical case mix in dogs (>50% of cases had multiple respiratory diagnoses, including congenital disorders causing cough) might have contributed to a reduction in statistical power, potentially explaining the absence of statistically significant findings	

SHS: secondhand smoke. BALF: bronchoalveolar lavage fluid.

* Last access 16 February 2025.

The last study, which failed to demonstrate an association between secondhand smoke and different respiratory diseases, enrolled both dogs and cats and investigated SHS as a component of indoor air pollution^32^. Exposure to SHS was assessed through a questionnaire completed by the owners; also, household PM 2.5 samples were collected in areas where animals spent most of the time as a proxy for indoor air pollution (SHS, incense burning, cooking). SHS was not significantly associated with respiratory diseases in dogs or cats, but in cats, there was a statistically significant association with PM2.5^32^.

One study found an unclear association between SHS and oral squamous cell carcinoma in 36 cats with a histologically confirmed diagnosis^33^. Cat owners completed a mailed questionnaire on 2-year pre-diagnosis exposures, investigating if the cat had ever lived in a household with one or more smokers, for how many years, and the average number of cigarettes smoked per day by all household members combined. The authors found that SHS exposure was associated with an approximately twofold increased risk of developing the neoplasm, but it was not statistically significant. However, cats living with owners smoking 1–19 cigarettes per day had a statistically significant fourfold increased risk, which was not confirmed in the ≥20 cigarettes per day category^33^.

Another study investigating oral squamous cell carcinoma (cytologically or histologically confirmed) in 100 cats found an approximately twofold increase in risk, which was statistically significant (p=0.03)^34^.

In 2017, Pinello et al.^35^ studied SHS exposure in 23 dogs, asking their owners to complete a questionnaire, and found a statistically significant association with lymphomas. Indeed, according to the number of Ki67 cells (as an index of cell proliferation), a significant positive association was found between the Ki67 index and the presence of smoking owners (p=0.002); furthermore, the index was also positively correlated with the number of smokers who lived with the dogs (p=0.001)^35^. A second study by the same author was conducted in 2025, investigating potential risk factors for canine non-Hodgkin lymphoma, including owners’ smoking habits^36^. A ‘composite smoking index’ (including secondhand and thirdhand smoke) was created by summing individual variables such as: presence of smokers in the household, number of smokers in the household, average daily cigarette consumption per smoker, presence of curtains, presence of carpets, frequent use of the fireplace, etc. The authors found that diseased dogs were exposed to a statistically significantly higher mean smoking index compared to the control group (p<0.001), whereas none of the factors related to tobacco exposure analyzed separately reached statistical significance^36^. A statistically significant association between SHS exposure and urinary bladder cancer (specifically urothelial carcinoma) was identified in a cohort of Scottish Terriers (p=0.033)^3^. The affected group not only exhibited a higher prevalence of residing in smoking households, but urothelial carcinoma was diagnosed in 35.5% of dogs with measurable urinary cotinine levels, compared to only 15.5% in those with undetectable cotinine concentrations (p=0.016)^3^. Another two studies focused on conditions other than respiratory diseases or neoplasia. The first study examined dogs affected by myxomatous mitral valve disease and found that individuals living in households with smokers exhibited statistically significantly lower plasma concentrations of tetrahydrobiopterin (p=0.01) [a cofactor for endothelial-derived vasodilator nitric oxide, whose depletion is associated with oxidative stress], suggesting that passive smoke exposure may contribute to oxidative imbalance^37^. The second study involved 20 cats exposed to SHS, alongside a control group, all presented for routine clinical examination and vaccination to a veterinary hospital^4^. Results indicated that SHS exposure negatively impacted oxidative status and promoted the release of pro-inflammatory cytokines. Specifically, total oxidant status (p<0.05), oxidative stress index (p<0.001), advanced oxidation protein products (p<0.001), and levels of IFN-γ (p<0.01), IL-1β (p<0.01), IL-2 (p<0.001), and IL-6 (p<0.01) were all elevated in exposed cats compared to controls. Additionally, serum creatinine and glucose levels were also increased in the SHS-exposed group (p<0.05), leading the authors to suggest a potential link between SHS exposure and increased susceptibility to renal disease and type II diabetes in cats^4^.

The last study investigated the possible association between chronic bronchitis and SHS exposure in dogs. Yamaya et al.^38^ investigated concentrations and methylation rates of free DNA fragments, as well as total nucleated cell and macrophage counts, in bronchoalveolar lavage fluid from 19 dogs, all with chronic bronchitis, divided into two groups (SHS-exposed vs non-exposed), classified according to serum cotinine levels^38^. SHS-exposed chronic bronchitis dogs had statistically significantly higher median values of inflammatory cells (total nucleated cells and macrophages) in comparison to non-SHS exposed dogs, but methylations rates and concentrations of DNA were not statistically significantly associated with SHS exposure^38^.

Finally, a review was also retrieved investigating potential etiologic factors for feline oral squamous cell carcinoma. Although some references reported a certain degree of association with SHS, the review concluded that the available evidence on the etiology of this neoplasm in cats is still limited^39^.

### Dogs and cats as environmental sentinels in the One Health paradigm

Dogs and cats have been investigated as potential environmental sentinels for passive smoke exposure, primarily through the analysis of specific biomarkers, such as cotinine (a nicotine metabolite and tobacco alkaloid) in various biological matrices (Table 3).

**Table 3:** Molecules investigated and found in different canine and feline substrate as markers of their exposure to tobacco smoke (International PubMed free research service for biomedical scientific literature)*

*Study number*	*Dogs*	*Cats*	*Investigated biomarkers*	*Substrate*	*Reference*
**1**	X	-	Cotinine	Serum, fur, and amniotic fluid	[5]
**2**	-	X	Nicotine, nicotine glucuronide, cotinine, cotinine glucuronide, and 4-(methylnitrosamino)-1-(3-pyridyl)-1-butanol (NNAL)	Urine	[40]
**3**	X	-	Cotinine	Urine	[41]
**4**	X	-	Cotinine	Serum and fur	[42]
**5**	X	X	30 aromatic amines, nicotine, and cotinine	Urine and feces	[43]
**6**	X	-	Cotinine	Ejaculate, serum and hair	[44]

* Last access 16 February 2025. Note: ‘X’ identify the species involved in the study; ‘-’ identify the species not involved in the study.

The presence of nicotine (p<0.001), cotinine (p<0.001), and of 4-(methylnitrosamino)-1-(3-pyridyl)-1-butanol (NNAL) (p=0.013) [a metabolite of the tobacco specific nitrosamine 4-(methylnitrosamino)-1-(3-pyridyl)-1-butanone] were detected in cats living with smokers, at statistically significantly higher concentrations than in cats from smoke-free households^40^.

Cotinine was also found at statistically significantly higher concentrations in canine urine of SHS-exposed dogs (p=0.02), concentrations increasing linearly depending on the number of cigarettes smoked by the household members (p=0.004)^41^. Cotinine levels also varied depending on the morphology of the dog’s muzzle, being higher (p=0.03) in short-nosed dogs compared to medium and long-nosed dogs^41^. Cotinine was also searched in other canine substrates such as serum and fur, as well as in the amniotic fluid of pregnant bitches. In the study of Groppetti et al.^42^, it was statistically significantly more present in serum and fur of SHS-exposed animals (p<0.001) (but it was also present in all non-exposed dogs), with higher levels in serum (p<0.001). Hair cotinine concentration was higher in females than in males (p<0.01), and it was not correlated to exposure levels; the article also provided cutoff values to discriminate between exposed and non-exposed dogs^42^. Investigation into the potential risks associated with SHS during pregnancy revealed the presence of cotinine in both pregnant dogs and their offspring. Cotinine was detected in serum, fur, and amniotic fluid samples from both SHS-exposed and non-exposed groups, with significantly higher concentrations observed in the exposed pregnant bitches (p=0.004)^5^.

Another study analyzed urinary and fecal concentrations of 30 aromatic amines (AAs), as well as cotinine and nicotine in pet dogs and cats. The findings revealed no significant association between the urinary or fecal levels of AAs and nicotine, suggesting that these compounds may originate from distinct sources^43^. Additionally, cotinine was detected in the semen of dogs cohabiting with both smoking and non-smoking owners, with statistically significantly higher levels observed in the former group (p=0.0002)^44^. Seminal cotinine concentrations were positively correlated with cotinine levels in blood and hair (p<0.0001)^44^. The study also investigated potential associations between SHS exposure and reproductive parameters, including total sperm concentration and total antioxidant capacity in semen, but found no significant differences between SHS-exposed and non-exposed dogs^44^.

Finally, two review articles were also retrieved, one published nearly three decades ago and the other more recently. Both reviews explored the role of pets as environmental sentinels^45,46^. They concur in recognizing dogs and cats as reliable indicators of environmental health risks relevant to humans.

### Further relevant evidence on passive smoke and pets

A case–control study investigating nasal cancer in dogs (103 cases and 378 controls with other cancer types) assessed associations with SHS by evaluating the number of cohabiting smokers, the number of cigarette packs smoked per day per smoker within the household, duration of smoking, and the time spent indoors by each dog. A significant association between SHS exposure and nasal cancer was observed specifically in dolichocephalic (long-nosed) breeds^47^. An earlier study identified an association between SHS exposure and lung cancer in brachycephalic (short-nosed) dogs^48^, while another study found DNA damage in the oropharyngeal tissues of dogs exposed to SHS^49^ suggesting a potential anatomical predisposition linked to muzzle morphology.

In cats, SHS has been identified as a potential risk factor for the development of malignant lymphoma, with a significant association observed between neoplastic risk and duration of exposure^50^. Additionally, both SHS exposure and its duration have been associated with p53 overexpression in feline oral squamous cell carcinomas, suggesting a possible role in tumorigenesis^51^. In dogs, prolonged exposure to cigarette smoke has been linked to various respiratory effects, including stimulation of the cough reflex, increased mucus production, bronchoconstriction, apnea, bradycardia, and hypotension. Furthermore, pathological changes in the lungs have been observed, resembling those found in human chronic bronchitis and emphysema, such as alveolar destruction, enlargement, and disruption of parenchymal tissue adjacent to the respiratory bronchioles^52^.

### E-cigarette and pets

Although the initial literature search identified 16 articles related to pets and electronic cigarette use, none was deemed relevant upon full-text review, a current gap in the literature.

## DISCUSSION

This scoping review aimed to update and synthesize current evidence regarding the health effects of SHS and e-cigarette aerosol exposure on the health of pets, particularly dogs and cats.

Despite some inconsistencies, particularly among studies assessing respiratory disorders and suffering from methodological limitations and possible biases^30-33^, the findings from the reviewed studies indicate a growing body of evidence supporting the harmful effects of SHS on pets, with reported associations ranging from respiratory and cardiovascular alterations to neoplasms and biochemical markers of oxidative stress and inflammation. Indeed, also evidence from human studies indicates that exposure to SHS is associated with a range of non-respiratory outcomes, including cardiovascular disease, urinary tract cancers, and immune dysregulation^9,14^.

In animals, certain anatomical features (e.g. muzzle length) and physiological factors (e.g. age, breed) appear to modulate vulnerability, with some studies suggesting increased susceptibility to specific disease types such as nasal or lung cancer depending on craniofacial conformation of dogs^41,47,48^.

Unfortunately, the small number of studies and differences in experimental designs present in the literature do not allow the present revision to clearly establish the effects of tobacco smoke exposure and the causal link, but in some cases, such as the feline oral squamous cell carcinoma, it is strongly suggested^34^. Potentially, all studies conducted, even in the absence of significant associations, deserve further investigation. Available studies suggest that pets are not only passive receivers of human environmental exposures, but may also function as biological indicators of home air quality^45,46^. Observed outcomes show that dogs and cats serve as effective sentinels for SHS exposure, and also reveal that they accumulate nicotine metabolites, particularly cotinine, in biological matrices such as urine, serum, fur, and even amniotic fluid, which may adversely affect their health^40-42^. Furthermore, several studies suggest that biomarkers could be useful for quantitatively assessing exposure. Many studies suffer from methodological limitations when reconstructing pets’ exposure to SHS using questionnaires, as responses may be influenced by owners’ perception of SHS and its potential harm to pets. In this context, the use of biomarkers may offer a more objective assessment. When exposure assessment relies on owner-completed questionnaires, the presence of fireplaces in the household should be taken into account, as they may serve as potential confounding factors in potential risk estimation^53,54^.

As previously reported, although e-cigarettes are claimed to be less dangerous than traditional smoking, they have both direct and indirect proven harmful effects^11-13^; therefore, it is plausible that secondhand aerosol exposure, increasing the body burden of toxic and carcinogenic chemicals, can cause health problems to dogs and cats too. No studies were found in the present search about e-cigarettes and canine and feline health; further research is definitely needed on this issue.

Of particular interest is the finding that pet owners may be more inclined to adopt smoke-free behaviors when made aware of the potential risk their smoking poses to their animals. Information about the harmful effects of SHS on pets may serve as an effective tool for primary tobacco prevention, by discouraging smoking initiation, and for secondary prevention, by helping to motivate smokers to reduce or quit cigarette use. The emotional bond between pet owners and their animals plays a pivotal role; studies have shown that many smokers, despite lacking motivation to quit for their own health, become more responsive and considerate when made aware that their behavior may harm their pets^55-57^. Milberger et al.^57^, in 2009, in a web-based survey to which participated 3293 adult pet owners, reported that providing information on the risks of cancer and respiratory diseases in animals increases pet owners’ willingness to adopt protective behaviors, such as quitting smoking or prohibiting smoking inside the home. Specifically, among pet owners who smoke, 28.4% stated that learning about the risks of SHS to their pets would motivate them to attempt quitting, while 8.7% would ask household members to stop smoking, and 14.2% would request that smoking not occur indoors. Furthermore, non-smoking pet owners living with smokers indicated that awareness of these risks would make them more likely to encourage cohabitants to quit smoking (16.4%) or to limit smoking to outdoor areas (24.2%).

These findings suggest that emphasizing the harmful effects of SHS on animals in tobacco control campaigns may serve as an innovative and effective educational strategy to promote smoking cessation, particularly among individuals who are less responsive to messages focused solely on human health. Educational initiatives that inform pet owners about the potential risks of SHS exposure could motivate some to quit smoking and encourage both smokers and non-smokers living with smokers to make their homes smoke-free.

Veterinary professionals can play an important role in raising awareness in pet owners about the potential health risk they expose their pets. Public health could include pet-focused messaging in anti-smoking campaigns as an effective behavioral motivator. These motivational factors may represent a novel and still underutilized angle in smoking cessation campaigns.

The absence of time restrictions in the literature search has clearly illustrated the societal shift in the perception of dogs and cats: from experimental subjects to valued family members (as well as environmental sentinels). In line with the One Health paradigm (an approach in which the protection of human health must be associated with the protection of animal health and of the environment), they deserve protection from environmental hazards, as it is evidenced by the evolution of national and international regulations concerning animal welfare and health. For instance, the Constitution of the Italian Republic, as amended in 2022, states in Article 9, paragraph 3: ‘The Republic shall safeguard the environment, biodiversity and ecosystems, also in the interest of future generations. State law shall regulate the methods and means of safeguarding animals’^58^. In line with Article 9 of the Italian Constitution, a new Italian law (No. 82; 6 June 2025) which came into force on 1 July 2025, establishes harsher penalties for those who mistreat or kill animals, with a particular focus on the protection of companion animals^59^. At the European level, Article 13 of the Consolidated Version of the Treaty on the Functioning of the European Union affirms the need to protect animal welfare, since ‘animals are sentient beings’^60^. These advancements in national and international regulations align with the One Health approach, which emphasizes the interconnection of human, animal, and environmental health.

### Strengths and limitations

Among the strengths of this review is its broad scope, encompassing both experimental and observational studies, and its explicit focus on integrating pets within the larger public health dialogue. The inclusion of biomarker-based research supports a more objective quantification of exposure and bridges the gap between environmental health monitoring and veterinary medicine. Studies employing biomarkers generally reported statistically significant results^3,4,38^. The findings also align with the One Health approach, underscoring the link between human and animal health in the context of shared environments.

Also several limitations must be acknowledged. Firstly, due to constraints, a systematic review was not possible, while the studies reviewed varied considerably in terms of methodology, sample size, objectives, exposure assessment methods and endpoints, limiting the comparability of results. In some cases, associations were observed but did not reach statistical significance, and only a few studies included appropriate control groups, thus also leading to differences in quality between eligible studies. Some studies enrolled small samples, and others included small numbers in subgroup analyses, which may explain the lack of statistically significant findings. Interestingly, summary measures were statistically significant, whereas individual tobacco exposure factors were not^33,36^. More studies involved dogs than cats, which is common in veterinary medicine, thus cats are under-represented. Another constraint is the reliance on owner-reported data in many studies, which may introduce recall and reporting bias and inaccuracies in exposure estimation. Moreover, some studies reported low response rates to mailed questionnaires, others enrolled fewer controls than cases, and some included a heterogeneous clinical case mix. These factors may have reduced statistical power, potentially explaining the absence of statistically significant findings^31,32^. Additionally, the lack of standardized exposure metrics (e.g. cotinine cutoff levels) may limit the generalizability of the findings. Furthermore, publication bias could also have occurred and potentially valuable data could have omitted because of the exclusion of non-English and non-open-access articles from the main inclusion criteria, or because of the possible presence of data published outside of the usual scientific channels as well as in the case of conference proceedings. However, some of these materials were retrieved and discussed to broaden the context.

Another consideration is the potential for residual confounding factors, such as the presence of indoor fireplaces or environmental pollutants unrelated to tobacco, which may influence health outcomes and complicate the attribution of effects solely to SHS.

The short lifespan of companion animals, while potentially increasing susceptibility to certain environmental burdens, also limits long-term observation.

Importantly, no eligible studies specifically addressed the effects of e-cigarette aerosol exposure on pets, despite growing use of these devices and evidence of their harm in humans^11-13^. This represents a critical gap in veterinary and environmental health research that deserves appropriate exploration.

### Future research

Future research should aim to homogenize as much as possible the design of the studies, so that they can be compared with each other, trying, if possible, to focus them on the main diseases (e.g. respiratory, neoplasm, CVD), standardizing exposure assessment methods, recommending the inclusion of large and more diverse populations, and exploring the effects of novel tobacco products and electronic cigarettes. Integrating veterinary and human medical data under the One Health framework could further enhance our understanding of shared environmental health risks and inform more effective prevention strategies.

Importantly, the emotional bond between pet owners and their animals presents a unique opportunity for targeted education campaigns. Studies have shown that many pet owners are more inclined to adopt protective behaviors - such as quitting smoking or enforcing smoking bans indoors - when they are made aware of the potential harm to their pets. This insight could inform future public health interventions aiming to reduce tobacco exposure in domestic environments.

## CONCLUSIONS

The current body of evidence suggests an association between SHS exposure and adverse health outcomes in dogs and cats. Pets may serve as effective sentinels of environmental risk, not only due to their close contact with human environments but also because of their demonstrated physiological responses to tobacco smoke. This recognition should prompt greater consideration of animal health in public health messaging and tobacco control strategies.

Beyond respiratory effects, evidence from human studies indicates that exposure to SHS is associated with a range of non-respiratory outcomes, including cardiovascular disease, urinary tract cancers, and immune dysregulation. These findings suggest that SHS exposure in companion animals may have multisystemic consequences and justify further research exploring outcomes beyond the respiratory system.

## Data Availability

Data sharing is not applicable to this article as no new data were created.
